# Sol-Gel Synthesis and Characterization of Yttrium-Doped MgFe_2_O_4_ Spinel

**DOI:** 10.3390/ma15217547

**Published:** 2022-10-27

**Authors:** Dovydas Karoblis, Kestutis Mazeika, Rimantas Raudonis, Aleksej Zarkov, Aivaras Kareiva

**Affiliations:** 1 Institute of Chemistry, Vilnius University, Naugarduko 24, LT-03225 Vilnius, Lithuania; 2 Center of Physical Sciences and Technology, LT-02300 Vilnius, Lithuania

**Keywords:** magnesium spinel, sol-gel synthesis, ferrite spinel, solid solutions, magnetic properties

## Abstract

In this study, an environmentally friendly sol-gel synthetic approach was used for the preparation of yttrium-doped MgFe_2_O_4_. Two series of compounds with different iron content were synthesized and A-site substitution effects were investigated. In the first series, the iron content was fixed and the charge balance was suggested to be compensated by a partial reduction of Fe^3+^ to Fe^2+^ or formation of interstitial O^2−^ ions. For the second series of samples, the iron content was reduced in accordance with the substitution level to compensate for the excess of positive charge, which accumulates due to replacing divalent Mg^2+^ with trivalent Y^3+^ ions. Structural, morphological and magnetic properties were inspected. It was observed that single-phase compounds can only form when the substitution level reaches 20 mol% of Y^3+^ ions and iron content is reduced. The coercivity as well as saturation magnetization decreased with the increase in yttrium content. Mössbauer spectroscopy was used to investigate the iron content in both tetrahedral and octahedral positions.

## 1. Introduction

Spinel ferrites with general formula MFe_2_O_4_ (where M = Fe, Co, Mn, Ni, Mg, Cu, Zn) are considered to be an important class of inorganic materials displaying a large variety of properties, including mechanical hardness, chemical stability, high electrical resistivity and good thermal stability [1]. In addition, these compounds are soft magnetic materials with low coercivity, high magnetization saturation and remnant magnetization at room temperature [2]. A variety of physical, electrical, dielectric, magnetic, optical and catalytic properties make ferrite spinels applicable in medicine [3], water and wastewater treatment [4], nonvolatile memory devices [5], catalysis [6], gas sensing [7], microwave absorption [8] etc.

Amongst all the spinel ferrites, magnesium ferrite (MgFe_2_O_4_) attracted considerable attention from the scientific community due to its high adsorption capacity, suitable bandgap and non-toxicity [9,10]. This compound has a partially inverse spinel structure, where divalent Mg^2+^ ions partly fill the tetrahedral site. It is known to be ferrimagnetic below Neel temperature; however, in the case of nanoscale particles depending on preparation conditions it can also be superparamagnetic [11,12]. Various synthesis approaches, such as co-precipitation [13], solvothermal [14], one-pot solution combustion [15], hydrothermal [16] and microwave-assisted ball milling [17] were utilized for the preparation of magnesium ferrite. Another widely applied synthesis technique is the aqueous sol-gel method [18,19,20]. Using different complexing agents allows the preparation of phase-pure magnesium ferrite spinel at a low processing temperature in a relatively short time. Moreover, the mixing of starting materials at the atomic level leads to the ease of doping with other elements [20].

One of the ways to tune the physical properties of spinel ferrites is doping with different monovalent, divalent or trivalent cations. For example, Mg^2+^ substitution by Zn^2+^ led to an increase in magnetization and magnetic moments [21]; the introduction of 80% of Co^2+^ ions into the MgFe_2_O_4_ structure resulted in the smallest core losses [22]. In particular, the effects of substitution by Y^3+^ ions were investigated for many ferrite spinels, including MnFe_2_O_4_ [23], Mn_0.5_Zn_0.5_Fe_2_O_4_ [24], CoFe_2_O_4_ [25], CdFe_2_O_4_ [26] and ZnFe_2_O_4_ [27]. In most cases, trivalent Fe^3+^ ions were substituted, while the amount of A-site cation remained the same. While yttrium has a larger ionic radius than other cations in the spinel crystal lattice, previous works [23,24,25,26,27] have shown that up to 30% can be successfully introduced into the ferrite spinel structure without the formation of any impurity phases.

To the best of our knowledge, there is only one work regarding MgFe_2_O_4_ spinel substituted with yttrium, where a double sintering technique was applied [28]. In this study, the introduction of Y^3+^ in favor of Fe^3+^ ions resulted in increased resistivity, decreased dielectric constant and reduced particle size. Additionally, it was shown that only 2 mol% of iron can be substituted since a higher yttrium amount resulted in the formation of the YFeO_3_ impurity phase.

In this work, we investigated Mg^2+^ substitution with Y^3+^ ions by preparing two different series of Mg_1−x_Y_x_Fe_2−δ_O_4_ powders applying an environmentally friendly sol-gel method. Since divalent ion is substituted by trivalent, in the first series (with δ = 0) the amount of Fe amount was fixed, while in the second (δ = x/3) the charge was compensated by an appropriately reduced amount of Fe. The structural, morphological and magnetic properties of the obtained spinels were evaluated. Moreover, the maximal yttrium substitution level, when monophasic compounds form, was also inspected.

## 2. Materials and Methods

For the preparation of yttrium-doped MgFe_2_O_4_ spinels, magnesium (II) nitrate hexahydrate (Mg(NO_3_)_2_∙6H_2_O, ≥98%, Chempur, Karlsruhe, Germany), iron (III) nitrate nonahydrate (Fe(NO_3_)_3_∙9H_2_O, 99.9%, Alfa Aesar, Haverhill, MA, USA) and yttrium (III) nitrate hexahydrate (Y(NO_3_)_3_∙6H_2_O, 99.9%, Sigma-Aldrich, St. Louis, MO, USA,) were used as starting materials. During the first step, the appropriate amounts of nitrates required for the synthesis of 1 g of material were dissolved in 20 mL of deionized water. After that, citric acid monohydrate (C_6_H_8_O_7_∙H_2_O, 99.9%, Chempur) and ethylene glycol (C_2_H_6_O_2_, ≥99.5%, Sigma-Aldrich) were added to the mixture (the molar ratio between total metal ions, citric acid and ethylene glycol was 1:1:2, respectively). The temperature of magnetic stirrer was set at 90 °C and the above solution was homogenized for 1 h under constant mixing. After that, the temperature was increased up to 120 °C for complete solvent evaporation and the gel was obtained. The acquired gel was left to dry overnight at 130 °C in the oven, carefully ground in agate mortar and annealed in air at 800 °C for 5 h with a heating rate of 5°/min. 

PerkinElmer STA 6000 Simultaneous Thermal Analyzer was used to perform thermogravimetric and differential scanning calorimetric (TG/DTG-DSC) analysis. A small amount of dried gel (5–10 mg) was heated at a 10 °C/min heating rate from 30 to 850 °C in dry flowing air (20 mL/min). Rigaku Miniflex II diffractometer using a primary beam Cu Kα radiation (*λ* = 1.541838 Å) was used for X-ray diffraction (XRD) analysis. The 2*Θ* angle of the diffractometer was selected in 20°–80° range while moving 5°/min. To calculate the crystallite size the Scherrer’s equation (*D* =$\;\frac{K\lambda }{\beta cos\Theta }$, where *K*—shape factor 0.89, *λ*—X-ray wavelength, *β*—full width at half maximum in radian, *Θ*—Bragg diffraction angle) was used. To determine the instrumental broadening, the *β* was measured for corundum standard. Fourier-transform infrared spectroscopy (FT-IR) was performed using an Alpha FT-IR spectrophotometer (Bruker, Ettlingen, Germany) in the range of 4000–400 cm^−1^. The morphology of solid solutions was examined using the Hitachi SU-70 (Tokyo, Japan) scanning electron microscope (SEM). Magnetometer consisting of the lock-in amplifier SR510 (Stanford Research Systems, Sunnyvale, USA), the gauss/teslameter FH-54 (Magnet Physics, Cologne, Germany) and the laboratory magnet supplied by the power source SM 330-AR-22 (Delta Elektronika, Zierikzee, The Netherlands) were applied to record magnetization dependences on the applied magnetic field. Mössbauer spectra were measured using ^57^Co(Rh) source and Mössbauer spectrometer (Wissenschaftliche Elektronik GmbH, Starnberg, Germany). For low temperature measurements, closed cycle He cryostat (Advanced Research Systems, Macungie, USA) was applied. One or two hyperfine field distributions, separate sextet and singlet/doublet were used to fit to Mössbauer spectra applying WinNormos Dist software. Isomer shift is given relative to α-Fe.

## 3. Results

**Figure 1:**
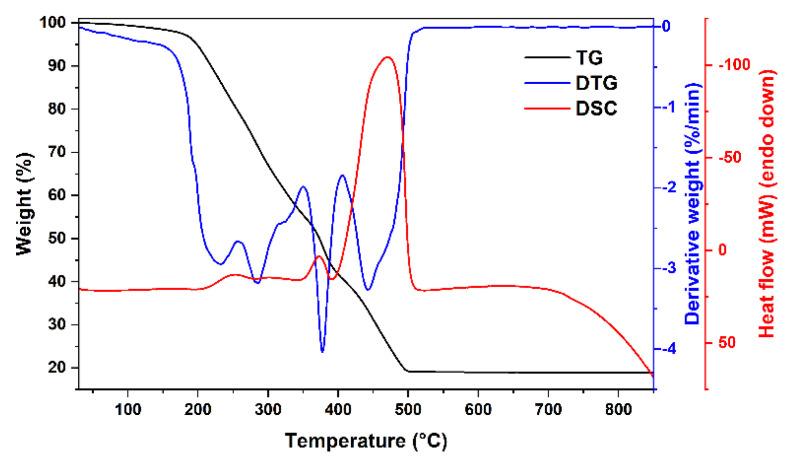
TG/DTG/DSC curves of Mg-(2)Fe-O xerogel.

**Figure 2:**
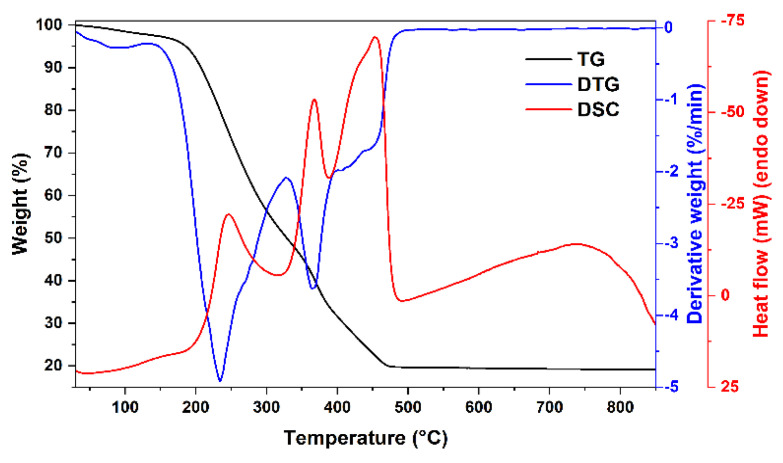
TG/DTG/DSC curves of (0.8)Mg-(0.2)Y-(1.933)Fe-O xerogel.

The thermal decomposition behavior of the obtained gels as well as possible minimal annealing temperature were investigated by thermogravimetric analysis. TG/DTG/DSC curves of two xerogels with different compositions (Mg-Fe-O and Mg-Y-Fe-O) are depicted in Figure 1 and Figure 2. The first degradation stage for both samples takes place in 50–130 °C range, where negligible weight loss (around 2%) can be seen. At these temperatures, the removal of adsorbed water occurs. Two degradation steps can be witnessed for Mg-Fe-O xerogel at 200–330 °C, while (0.8)Mg-(0.2)Y-(1.933)Fe-O xerogel has only one in this temperature range. These decomposition steps (where around 50% of the initial weight was lost) could be related to the decomposition of metal complexes with ethylene glycol and citric acid. A small exothermic peak found in DSC curve at around 250 °C supports the combustion reaction. Moreover, the next step centered around 370 °C in the DTG curve for both xerogels could be attributed to the thermal decomposition of metal nitrates and organic species. The last degradation step, in the 440–500 °C range for Mg-Fe-O xerogel and 380–470 °C range for (0.8)Mg-(0.2)Y-(1.933)Fe-O is related to pyrolysis as well as combustion of intermediates species formed during gelation and residual organic parts. Both xerogels lose around 80 °C of total weight according to TG curves. Interestingly, the sample containing 20 mol% of yttrium has a lower decomposition temperature compared to the sample with only magnesium and iron ions. 

**Figure 3:**
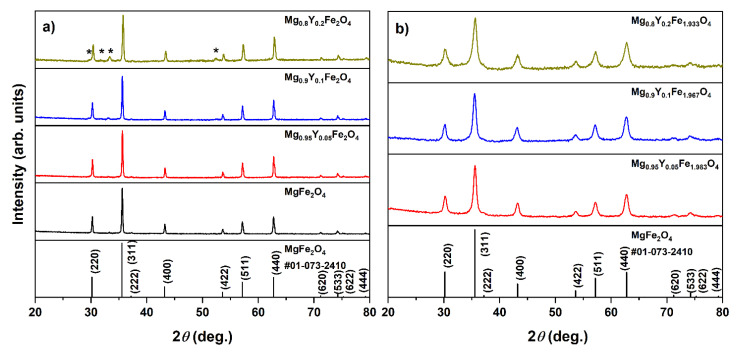
XRD patterns of Mg_1−x_Y_x_Fe_(2-−δ)_O_4_, where δ = 0 (**a**) and δ = x/3 (**b**). An asterisk represents the diffraction peaks ascribed to YFeO_3_ impurity phase.

While 460 °C was determined to be the lowest possible annealing temperature for the synthesis of Mg_0.8_Y_0.2_Fe_1.933_O_4_ spinel, only 800 °C temperature was sufficient for the formation of the spinel phase. Two different compositions of yttrium-doped spinels were prepared at this temperature and the results of the X-ray diffraction analysis are presented in Figure 3.

The undoped MgFe_2_O_4_ sample seems to be nearly monophasic with only a negligible amount of Fe_2_O_3_ as a neighboring phase. The phase purity of solid solutions depends on the iron content. In the first case, when Mg^2+^ was substituted with Y^3+^ ions and the iron content remained fixed throughout the whole series (Figure 3a), the formation of the hexagonal YFeO_3_ (#00-048-0529) impurity phase occurred. The amount of neighboring yttrium ferrite phase increased with the increase of Y^3+^ content. For the second series of samples (Figure 3b), since Mg^2+^ and Y^3+^ ions have different valencies, the excess of positive charge was compensated with appropriate reducing of the Fe^3+^ content. Mg_1−x_Y_x_Fe_(2−δ)_O_4_ (δ = x/3) solid solutions were monophasic until x = 0.2. With the increase of yttrium content, these materials demonstrated considerably broader diffraction peaks, indicating the formation of smaller particles. The crystallite size decreased from ca. 40 nm for MgFe_2_O_4_ to 10–13 nm for yttrium-containing solid solutions. This effect can be assumed as evidence of the introduction of yttrium ions into the crystal lattice. Moreover, only a slight shift of diffraction peaks to lower 2Θ can be seen, due to the difference in ionic radii between magnesium and yttrium ions (0.72 Å vs. 0.9 Å in VI-fold coordination) [29]. It should be noted that yttrium ions may also be located in Fe-sites at the octahedral position, since the iron amount in Mg_1−x_Y_x_Fe_(2-−δ)_O_4_ (δ = x/3) solid solutions was also reduced. While the orthorhombic YFeO_3_ phase is considered to be a thermodynamically stable one, the hexagonal perovskite phase can be prepared with a similar sol-gel methodology at lower temperatures [30,31]. It can be summarized that the compensation of excess of positive charge by reducing iron content was crucial for the preparation of single-phase spinel ferrites.

**Figure 4:**
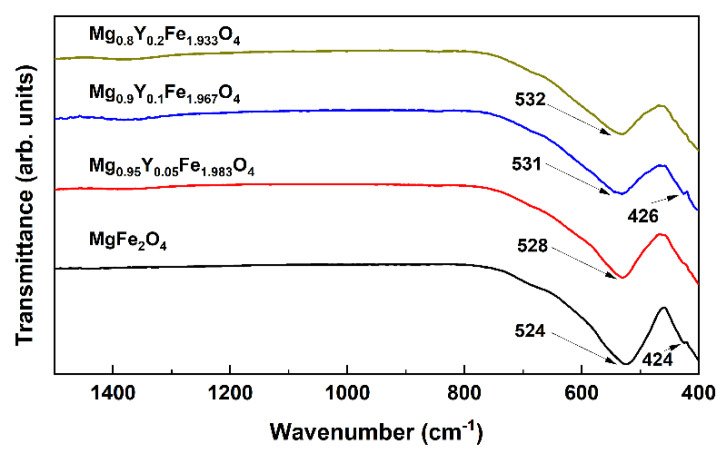
FT-IR spectra of Mg_1−x_Y_x_Fe_(2-−δ)_O_4_ (δ = x/3) specimens.

FT-IR spectroscopy was additionally performed to investigate the structural changes caused by the introduction of Y^3+^ ions into the spinel structure; the spectra of Mg_1−x_Y_x_Fe_(2-−δ)_O_4_ (δ = x/3) solid solutions are demonstrated in Figure 4. Since a relatively high annealing temperature was used for the preparation of ferrite spinels, no absorption bands were observed in the 4000–800 cm^−1^ range, which could be related to hydroxide, carbonate or residual organic species. According to the previous study of various spinel ferrites [32], the FT-IR spectrum of MgFe_2_O_4_ contains two bands assigned to the Fe-O bond centered at 565 and 406 cm^−1^. The first band is associated with the intrinsic vibrations in the tetrahedral site, while the latter is attributed to octahedral groups. In our case, the position of absorption bands is slightly different, which could indicate a redistribution of cations between A and B sites. A similar inversion of cations between both sites was previously observed for nanosized MgFe_2_O_4_ spinel prepared via sol-gel synthesis technique [33]. The absence of change in the position of the lower intensity band and monotonous, but a non-significant shift of the most intense absorption band with increasing yttrium content could suggest that Y^3+^ ions occupy tetrahedral positions in the lattice. 

**Figure 5:**
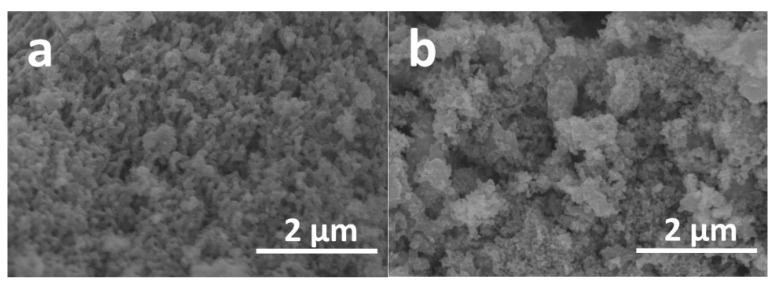
SEM images of Mg_0.95_Y_0.05_Fe_1.983_O_4_ (**a**) and Mg_0.9_Y_0.1_Fe_1.967_O_4_ (**b**) solid solutions.

Scanning electron microscopy (SEM) was employed to evaluate surface morphology as well as the particle size of two different yttrium-containing solid solutions and the results are presented in Figure 5. In both cases, samples consist of smaller particles, which are connected to each other forming larger aggregates. For Mg_0.95_Y_0.05_Fe_1.983_O_4_ sample (Figure 5a) the size of these aggregates varied in the 200–400 nm range, while for the sample containing 10 mol% of yttrium (Figure 5b), assemblies of particles were larger (200–600 nm). Interestingly, while the increase in Y^3+^ content resulted in the formation of larger aggregates, for individual particles the opposite effect can be seen. ImageJ was used to estimate the size of separate particles for both solid solutions, and most particles of Mg_0.9_Y_0.1_Fe_1.967_O_4_ spinel lay in the range of 30–100 nm, while Mg_0.95_Y_0.05_Fe_1.983_O_4_ sample was comprised of slightly larger particles varying from 50 to 150 nm. 

**Figure 6:**
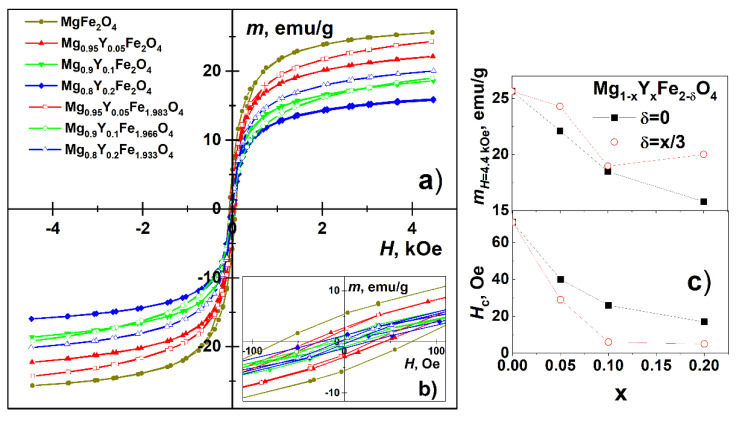
Magnetization hysteresis loops of Mg_1−x_Y_x_Fe_2−δ_O_4_ (**a**,**b**) and their parameters (**c**): saturation magnetization m_H = 4.4 Oe_ and coercivity H_c_.

With an increase of Y amount the saturation magnetization (at maximal applied field) of hysteresis loops of Mg_1−x_Y_x_Fe_2−δ_O_4_ (Figure 6) decreased from 26 emu/g to 16 emu/g (δ = 0) or 20 emu/g (δ = x/3), while the coercivity decreased from 70 to 17 Oe (δ = 0) or 5 Oe (δ = x/3). Cations in Mg ferrite are distributed between tetrahedral A and octahedral B sublattices which are denoted in formula (Fe_α_Mg_1−α_)[Fe_2−α_Mg_α_]O_4_ by round and square brackets, respectively. The cation redistribution is also known for other compounds with spinel structures, such as MgAl_2_O_4_ [34]. The Mg ferrite inversion degree is high (α ≈ 0.9) with Mg occupying predominantly octahedral sublattice [35,36,37]. The magnetization of Mg ferrite is determined by the difference in magnetic moments of Fe in tetrahedral and octahedral sublattices. By changing the chemical composition, Fe cations may redistribute between A and B sublattices of Mg_1−x_Y_x_Fe_2−δ_O_4_ in a way causing a change in magnetization. However, the major factor causing a decrease in magnetization could be a decrease in grain (nanoparticle) size and an increase in the contribution of a magnetically disordered, magnetically dead intergranular layer. The decrease in coercivity can also be explained by the formation of smaller superparamagnetic nanograins [38].

**Figure 7:**
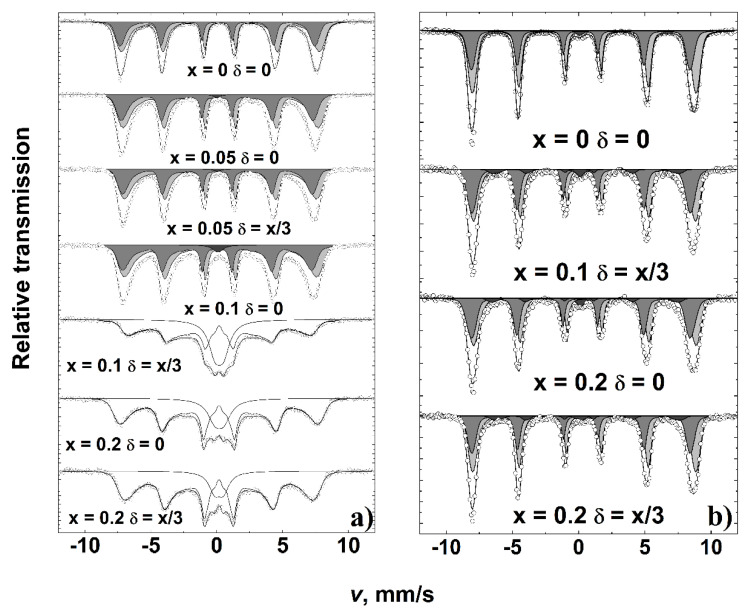
Mössbauer spectra of Mg_1−x_Y_x_Fe_2−δ_O_4_ at 296 K (**a**) and 10 K (**b**).

Mössbauer spectra of the MgFe_2_O_4_ sample (Figure 7a) showed broader spectral lines as compared to those of previously studied polycrystalline Mg ferrite [35,36]. The broadening of room temperature Mössbauer spectra and the relative area of superparamagnetic doublet increased with an increase of Y^3+^ content (Table 1). Two broad overlapping subspectra expressed by hyperfine field distribution P(B) attributable to octahedral A and tetrahedral B sublattices were distinguished by different isomer shifts: δ_A_ ≈ 0.15 mm/s and δ_B_ ≈ 0.4 mm/s for Mg_1−x_Y_x_Fe_2−δ_O_4_ samples with x ≤ 0.1. However, at larger spectral broadening, when x ≥ 0.1, only one P(B) distribution having an isomer shift of ≈0.30 mm/s was used, which was an average isomer shift of two A and B subspectra. The isomer shift ≈ 0.30 mm/s was characteristic of the superparamagnetic doublet. The decrease in average hyperfine field and increase of doublet area can be explained by the increase of the superparamagnetic relaxation rate of Fe spins in case of a decrease in grain size. The average hyperfine field of the whole spectrum <B_all_> decreased up to 50% (Table 1) with an increase of x. It was the smallest for Mg_0.9_Y_0.1_Fe_1.967_O_4_ having the largest contribution of doublet in the spectrum. It can be noted that Mössbauer spectra do not indicate that Fe^2+^ ions may form as there were no characteristic shifts of spectral shape while increasing the substitution of Mg^2+^ by Y^3+^.

At 10 K the width of hyperfine distributions of two major subspectra shrank (Figure 7b). The additional subspectrum of 5% spectral area (Table 2) was distinguished for yttrium-containing samples. The additional subspectrum with an average hyperfine field of 42–43 T can be attributed to Fe-disordered sites because of Y presence in the neighborhood of Fe sites or the formation of hexagonal YFeO_3_. We were not able to find previously published low-temperature Mössbauer data for hexagonal YFeO_3._ However, for Fe in hexagonal YMnO_3_, the positions of the lines in the Mössbauer spectrum (hyperfine field of 40–44.5 T) at 12 K [39] are in rather good agreement with those of additional subspectra.

## 4. Conclusions

A sol-gel synthetic approach using ethylene glycol and citric acid was successfully utilized for the preparation of Mg_1−x_Y_x_Fe_2−δ_O_4_ (δ = x/3) solid solutions. The iron content played a key role in the phase purity of the final products and only when δ = x/3 monophasic spinels could be obtained. At fixed iron content (δ = 0), the formation of a secondary YFeO_3_ phase occurred. While the size of individual particles was smaller with the increase in yttrium amount, the size of aggregates was larger. Intercalation of Y^3+^ ions caused a decrease in the saturation of magnetization and coercivity. According to the Mössbauer spectroscopy studies, with the increase in yttrium amount for Mg_1−x_Y_x_Fe_2−δ_O_4_ solid solutions the amount of iron located in the tetrahedral position increased. Low-temperature Mössbauer measurements revealed the formation of hexagonal YFeO_3_ or disordered phase.

## Figures and Tables

**Table 1:** Mg_1−x_Y_x_Fe_2−δ_O_4_ Mössbauer spectra parameters at 293 K: average hyperfine field <B>, subspectra A and B area ratio I_A_/I_B_, singlet relative area I_s_, isomer shifts δ. Indexes A, B and s indicate tetrahedral, octahedral sublattices and singlet, respectively.

x	δ	<B_A_>, T	<B_B_>, T	I_A_/I_B_	I_s_, %	δ_A_, mm/s	δ_B_, mm/s
0	0	45.0	45.0	0.62	0	0.16 ± 0.01	0.39 ± 0.01
0.05	0	41.6	43.4	0.7	1	0.14 ± 0.01	0.40 ± 0.01
0.05	0.017	41.2	42.3	0.7	2	0.16 ± 0.01	0.40 ± 0.01
0.1	0	39.6	40.8	0.74	1	0.15 ± 0.01	0.40 ± 0.01
		<B_AB_>, T	<B_all_>, T			δ_AB_, mm/s	δ_s_, mm/s
0.1	0.034	31.4	21.8	-	31	0.30 ± 0.01	0.31 ± 0.01
0.2	0	35.3	28.4	-	20	0.29 ± 0.01	0.31 ± 0.01
0.2	0.067	34.1	29.4	-	14	0.29 ± 0.01	0.29 ± 0.01

**Table 2:** Mg_1−x_Y_x_Fe_2−δ_O_4_ Mössbauer spectra parameters at 10 K: subspectra relative intensity I, isomer shift δ, quadrupole shift 2ε and average hyperfine field <B>.

x	δ	I, %	δ, mm/s	2ε, mm/s	<B>, T	
0	0	37	0.27 ± 0.01	0.01 ± 0.01	51.3	Tetrahedral A
		63	0.51 ± 0.01	0.05 ± 0.01	52.3	Octahedral B
0.1	0.034	34	0.27 *	0.10 ± 0.01	51.0	Tetrahedral A
		61	0.51 *	−0.01 ± 0.01	51.7	Octahedral B
		5	0.39 ± 0.02	0.54 ± 0.05	42.7	Disordered/h-YFeO_3_
0.2	0.067	36	0.27 ± 0.01	0.03 ± 0.01	51.2	Tetrahedral A
		59	0.51 ± 0.01	0.03 ± 0.01	51.9	Octahedral B
		5	0.45 ± 0.02	0.63 ± 0.05	42.6	Disordered/YFeO_3_
0.2	0	35	0.27 *	0.12 ± 0.01	51.2	Tetrahedral A
		60	0.51 *	0.01 ± 0.01	51.7	Octahedral B
		5	0.33 ± 0.03	0.55 ± 0.07	43.4	Disordered/YFeO_3_

* Fixed.
